# Response Surface Methodology for Optimization of Ultrasound-Assisted Antioxidants Extraction from Blackberry, Chokeberry and Raspberry Pomaces

**DOI:** 10.3390/plants13081120

**Published:** 2024-04-17

**Authors:** Iga Piasecka, Rita Brzezińska, Stanisław Kalisz, Artur Wiktor, Agata Górska

**Affiliations:** 1 Department of Chemistry, Institute of Food Sciences, Warsaw University of Life Sciences, 166 Nowoursynowska Street, 02-787 Warsaw, Poland; rita_brzezinska@sggw.edu.pl (R.B.); agata_gorska@sggw.edu.pl (A.G.); 2 Department of Food Technology and Assessment, Institute of Food Sciences, Warsaw University of Life Sciences, 166 Nowoursynowska Street, 02-787 Warsaw, Poland; stanislaw_kalisz@sggw.edu.pl; 3 Department of Food Engineering and Process Management, Institute of Food Sciences, Warsaw University of Life Sciences, 166 Nowoursynowska Street, 02-787 Warsaw, Poland; artur_wiktor@sggw.edu.pl

**Keywords:** ultrasound-assisted extraction, by-products, pomace, response surface methodology, polyphenols

## Abstract

An investigation of the ultrasound-assisted extraction (UAE) of polyphenol-rich aqueous extracts from blackberry, chokeberry and raspberry pomaces was carried out. The aim of the study was to choose optimal conditions for UAE in order to obtain extracts rich in phenolic compounds. The optimization was carried out based on response surface methodology. The variable conditions were amplitude of ultrasound wave and extraction time, whereas responses were total polyphenol content and antioxidant capacity. Based on the ANOVA analysis, mathematical models were fitted and verified. The most effective conditions of amplitude and time were 98% and 5.00 min, 78% and 10.32 min and 90% and 11.56 min for blackberry pomace, chokeberry pomace and raspberry pomace, respectively. The actual results obtained in optimized conditions were comparable to the results predicted by the models. Additionally, the anthocyanin content in extracts was determined in the high-performance liquid chromatography assay. It was proven that response surface methodology could be a useful tool in the optimization of UAE processes for obtaining polyphenol-rich extracts from berry fruit pomaces.

## 1. Introduction

Pomace is a main by-product of juice, concentrate or wine production. Conventional upcycling procedures include using pomaces as animal feed or fertilizer [1]. However, they are still abundant in antioxidants, like anthocyanins, phenolic acids, flavanols, etc. [2,3]. The recovery of phenolic compounds may be beneficial since it adds value to the side streams of fruit production and saves useful ingredients from being wasted. To maximize the benefits of antioxidant recovery from pomaces while minimizing environmental impact, the separation method should be carefully selected. Conventional extraction methods including solid–liquid extraction are characterized by their high consumption of time and organic solvents. Also, they are not always sufficiently effective. To reduce the aforementioned adverse effects, alternative isolation methods such as ultrasound-assisted extraction (UAE) could be taken into consideration. UAE utilizes the phenomenon of collapsing cavitation bubbles, which causes local events in the extracted material and in the liquid solvent. These events include intensified transportation of matrix compounds to the liquid, accelerated penetration of the solvent into the plant material and the fragmentation or erosion of solid material [4]. The advantages of using UAE instead of conventional extraction methods include higher extraction yields, decreased processing time and higher selectivity (depending on relevant solvent selection) [5]. To classify UAE as a fully green method, green solvents have to be selected as well. In the case of phenolic compounds, the most popular solvents are organic short-chain alcohols, like ethanol [6]. However, their use poses a risk of food contamination, which is why water was chosen as a solvent in the present study.

The variety of possible conditions for UAE is large, as the time, ultrasound amplitude, temperature, solvent, solid-to-liquid ratio, etc., are all modifiable. To reduce the number of experiments and optimize the process, response surface methodology (RSM) seems to be a useful tool. Briefly, RSM is composed of statistical and mathematical methods based on the fit of a polynomial model to the data in order to generate statistical predictions. It is used to optimize processes where the results (responses) are dependent on several variables [7]. It has been successfully employed in the optimization of the UAE of bioactive compounds from carrot pomace [8], blueberry pomace [9], apple pomace [10], grape pomace [11] and apricot pomace [12].

The main aim of the following study was to optimize the ultrasound-assisted extraction of polyphenols from blackberry (BB), chokeberry (CH) and raspberry (RB) pomaces using response surface methodology. The central composite design of the experiment was established in order to achieve the highest possible total polyphenol content (TPC) and antioxidant capacity measured spectrophotometrically. Then, the best parameters chosen were checked to determine whether the solution predicted by the software is actually well-fitted. Additionally, the extracts obtained in the optimized conditions and control extracts were subjected to anthocyanin content and profile analyses using high-performance liquid chromatography. Also, the correlation between the TPC and antioxidant capacity was studied.

## 2. Results and Discussion

### 2.1. Total Polyphenol Content

The TPCs of aqueous pomace extracts were measured through a colorimetric assay using spectrophotometry and Folin–Ciocalteu reagent. The used method is fast and simple—the color intensity is correlated with the concentration of reducing compounds, e.g., polyphenols. The reaction is not specific, and as a result, total polyphenol content estimation may be determined [13].

**Figure 1:**
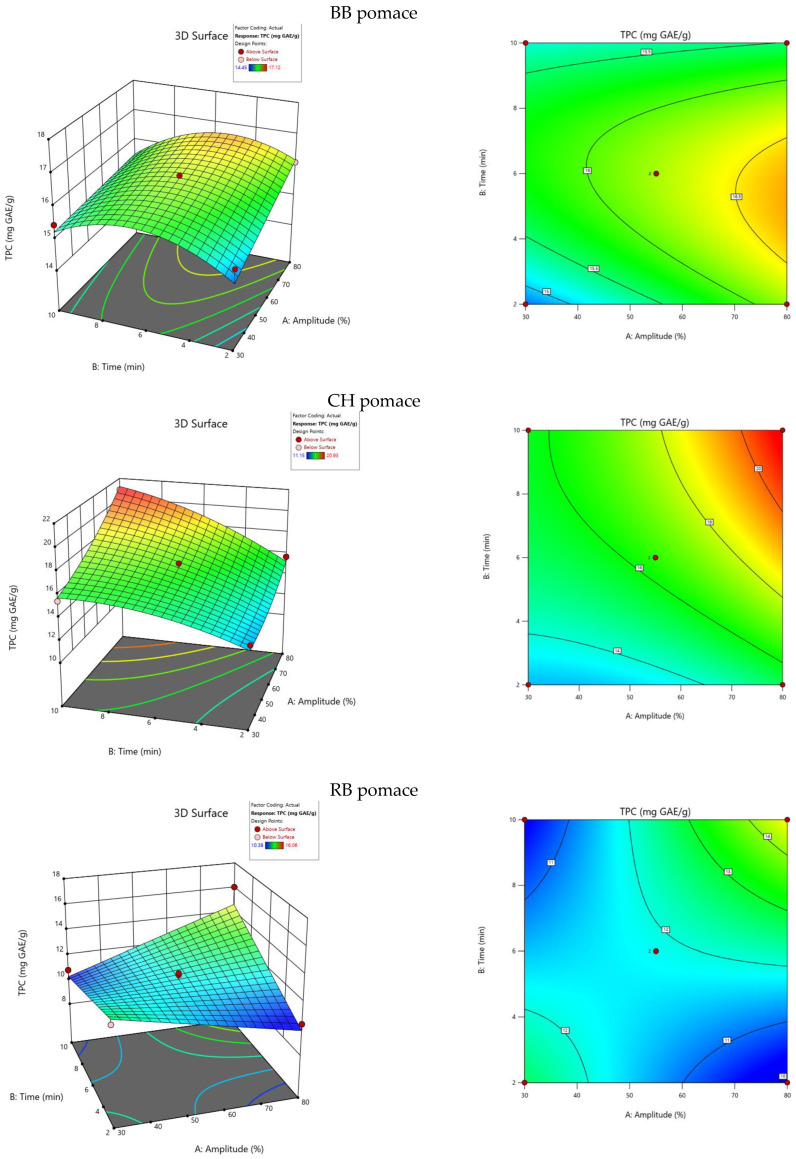
Three-dimensional and contour graphs showing total polyphenol content dependence from extraction time and ultrasound amplitude.

The effects of the ultrasound amplitude (X_1_) and extraction time (X_2_) variables on the TPC were analyzed based on 10 experimental runs carried out in 3 repetitions for each pomace. The specific TPC values obtained in the experiment were in the ranges of 14.62–17.12 mg GAE/g, 13.39–20.93 mg GAE/g and 10.38–16.06 mg GAE/g for BB, CH and RB pomace extracts, respectively. The response surface graphs of the experimental results were shown in Figure 1. There are visible surfaces with more effective results in terms of TPC. Briefly, the application of higher amplitude levels resulted in higher TPCs in all of the extracts. In terms of extraction time, CH and RB pomace extracts obtained through longer-lasting extraction procedures had higher TPCs; for BB pomace extract, moderate extraction times were the most effective.

The results of TPC analyses constituted the basis of the fitting of the mathematical models, described by the following equations (Equations (1)–(3)):

(1) $$TPC_{BB}=12.61+0.03\cdot X_{1}+0.75\;\cdot X_{2}-0.003\cdot X_{1}X_{2}+0.000001\cdot {X_{1}}^{2}-0.05\cdot {X_{2}}^{2}$$

(2) $$TPC_{CH}=12.70-0.08\cdot X_{1}+0.7\cdot X_{2}+0.008\cdot X_{1}X_{2}+0.001\cdot {X_{1}}^{2}-0.05\cdot {X_{2}}^{2}$$

(3) $$\;TPC_{RB}=16.02+-0.09\cdot X_{1}-0.84\;\cdot X_{2}+0.02\cdot X_{1}X_{2}$$

where X_1_ is the ultrasound amplitude and X_2_ is the extraction time.

Based on the ANOVA analysis, mathematical models were verified (Table 1). For BB and CH pomace extracts, the quadratic model was significant (at *p* < 0.05), with an insignificant (*p* > 0.05) lack of fit. RB pomace extraction was described with a two-factorial model (*p* < 0.05), and the lack of fit was not significant (*p* > 0.05). For all models, ANOVA results indicated relatively high F-values, which means that models were fitted with proper accuracy.

Generally, the higher the ultrasound amplitude is, the greater the compounds’ outflow into the liquid medium is improved. That is why TPC values in higher amplitudes were improved. However, acoustic cavitation effects also include rapid events, like collapsing cavitation bubbles, which results in local increments in temperature and pressure. These desirable effects accelerate the mass transfer, but can also be responsible for the degradation of thermolabile compounds, like polyphenols [14]. Thus, prolonged extraction times may result in decreases in the TPC.

The obtained results are similar to some previously published by other authors. Kaur et al. [15] found that moderate UAE times but high US power were the most efficient conditions for Java plum pomace extraction. Liao et al. [16] described the time effect of UAE on the TPC of eggplant peel extract. The TPC increased gradually as the time prolonged, but after reaching a critical time point—35 min in this case—the TPC started to decrease. However, some researchers have reported the opposite effect of time. Blueberry and raspberry extracts obtained using UAE over 45 min were characterized by higher TPC values than extracts obtained with a UAE time of 15 min using the same US power [17]. Silva Junior et al. [18] concluded that applying the longest time (15 min) and the highest value of ultrasound amplitude (100%) resulted in ciriguela peel extracts with the highest TPC results.

### 2.2. Antioxidant Capacity

The antioxidant capacity of pomace extracts was measured using radical-2,2′-azino-di-[3-ethylbenzthiazoline sulfonate (6)] (ABTS). This assay is based on the reduction of radical cations by bioactive compounds. In particular, the color intensity of the ABTS solution measured spectrophotometrically decreases in the presence of antioxidants [19].

**Figure 2:**
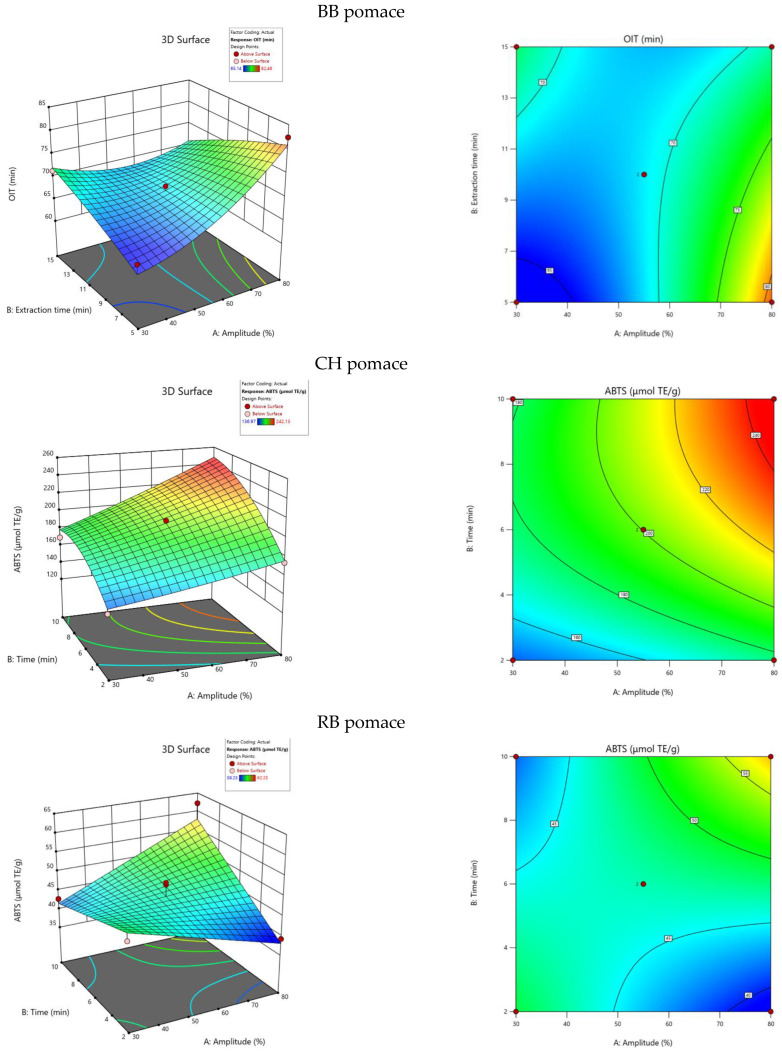
Three-dimensional and contour graphs showing antioxidant capacity dependence from extraction time and ultrasound amplitude.

Similarly to the TPC, the impact of ultrasound amplitude and extraction time on the antioxidant capacity of extracts was investigated based on 10 experimental runs repeated thrice for BB, CH and RB pomaces. The results of the assays varied in ranges of 26.21–109.21 μmol TE/g, 142.03–242.13 μmol TE/g and 39.23–62.25 μmol TE/g for BB, CH and RB, respectively. The response surface 3D and contour graphs were shown in Figure 2. The antioxidant capacity of the studied pomaces follows a similar trend as the TPC. The application of higher amplitude values was connected to increased antioxidant capacity results. In the cases of the CH and RB pomace extracts, longer extraction times resulted in higher antioxidant activities as well. However, in contrast, BB pomace extract obtained in a shorter time had higher antioxidant capacity values. The antioxidant capacity results are directly correlated with the TPC results [20] and are due to the same phenomena occurring during UAE as were explained for the TPC dependencies.

Equations (4)–(6) describe the mathematical dependence of the ABTS assay results on the applied ultrasound amplitude and extraction time. They reflect the fitting of the values to the proper statistical models:

(4) $$ABTS_{BB}=-27.65+0.77\cdot X_{1}+18.66\;\cdot X_{2}-0.05\cdot X_{1}X_{2}+0.002\cdot {X_{1}}^{2}-1.32\cdot {X_{2}}^{2}$$

(5) $$ABTS_{CH}=116.3+0.07\cdot X_{1}+11.83\cdot X_{2}+0.1\cdot X_{1}X_{2}+0.003\cdot {X_{1}}^{2}-0.92\cdot {X_{2}}^{2}$$

(6) $$\;ABTS_{RB}=62.07-0.36\cdot X_{1}-3.04\;\cdot X_{2}+0.07\cdot X_{1}X_{2}$$

where X_1_ is the ultrasound amplitude and X_2_ is the extraction time.

The ANOVA test results for the models’ fitting are summarized in Table 2. For the antioxidant capacity of ultrasound-extracted BB and CH pomaces, the quadratic model was significant (*p* < 0.05) with a not significant result for lack of fit (*p* > 0.05). The antioxidant capacity of RB pomace extract obtained via the use of ultrasound was described as significant using the two-factorial model (*p* < 0.05) and had an insignificant lack of fit (*p* > 0.05). High R^2^ and F-values of the models indicate proper model fitting and their significance.

In the study by Mazza et al. [21], the antioxidant capacity (ABTS) of grape skin extract also increased with the increasing ultrasound power. Similarly to the CH and RB pomace extracts, Anticona et al. [22] observed higher antioxidant activity, measured in DPPH and ABTS assays when a longer extraction time was applied (30 min). Results comparable to those of the BB pomace extract’s antioxidant capacity were obtained for the UAE of banana peel extract [23]. The highest values for the ABTS assay were observed when high ultrasound power was applied in a short time—250 W and 5 min, respectively.

### 2.3. Optimization

Based on the fitted mathematical models and their equations, the optimal parameters were determined using statistical software. The ultrasound amplitude taken into consideration for optimization was set in a range of 20–100%, the time was set as in a range of 2–12 min and the responses were set as high as possible. As a result, one solution with the highest desirability for each pomace extraction was chosen. Table 3 shows the optimal conditions suggested by the software with actual conditions applied, as the ultrasound processor has some limitations in terms of setting the ultrasound amplitude. The predicted results of responses are also specified—namely, the TPC and ABTS. Comparing to the experimentally obtained TPC and ABTS results, it can be observed that the model predictions were adequate, and all the results were within the 95% confidence interval.

Previously conducted optimization of polyphenols obtained via UAE from blackcurrant and redcurrant pomaces revealed that the optimal ultrasound amplitudes and times were 51% in 3 min and 91% in 11 min for blackcurrant and redcurrant, respectively [24]. Silva et al. [25] optimized the ethanolic UAE of polyphenols from acerola waste and a reported similar extraction time—13.6 min—to be the most efficient in terms of TPC, total flavonoid content and antioxidant capacity, as measured in a DPPH assay. The UAE of BB pomace with water was previously optimized by Zafra-Rojas et al. [26]. The chosen optimal conditions were similar to the values obtained in the present study. Researchers stated that a 91% amplitude and 15 min extraction time were the most effective. Applying those conditions, a TPC equal to 12.01 mg GAE/g and an antioxidant capacity (ABTS) equal to 63.19 μmol TE/g were obtained. Also, dos Santos et al. [27] described similar results for the optimization of aqueous UAE of BB pomace. In their findings, a 40% amplitude in 10 min and a solid concentration of 25 mg/mL were the optimal conditions. Their application resulted in a TPC of 44.12 mg GAE/g. The optimization of CH pomace extraction using ultrasound and ethanol as a solvent was conducted by Ramić et al. [28]. The optimal conditions in terms of the highest TPC value (15.41 mg GAE/mL of extract) were an ultrasonic power, temperature and time of 206.64 W, 70 °C and 80.1 min, respectively. However, as the design of that study was slightly different than that of the present study, it is not possible to credibly compare their results with those from the present study. The optimization of RB pomace extraction was conducted by Xue et al. [29]; however, they applied an ultrasound-assisted enzyme extraction procedure. The optimal parameters were an ultrasound power, temperature, pectinase dosage and extraction time of 290 W, 44 °C, 0.16% and 30 min, respectively.

### 2.4. Anthocyanin Content

Extracts obtained using the optimal ultrasound treatment conditions were analyzed for their anthocyanin content using an HPLC assay. The results of the HPLC assay are summarized in Table 4. Chokeberry pomace extract stands out as the most abundant in anthocyanins, especially cyanidin-3-galactoside. Extracts from blackberry and raspberry pomaces were characterized by over 2-fold lower total anthocyanin contents (TACs) than extracts obtained from chokeberry pomace. Concerning anthocyanins identified in blackberry pomace extract, cyanidin-3-glucoside represented over 91%. In raspberry pomace extract, cyanidin-3-sophoroside was the most abundant anthocyanin. The anthocyanin profiles of pomaces are dependent on the fruit variety, cultivation method and conditions.

In the study by Jara-Palacios et al. [30], the most abundant anthocyanin in blackberry pomace extract was also cyanidin-3-glucoside, with an 86% share in the TAC. However, in contrast to our study, another identified anthocyanin was cyanidin-3-rutinoside, which was not detected in the present work. Considering chokeberry pomace extract, cyanidin-3-galactoside was identified as major anthocyanin in studies by Rodríguez-Werner et al. [31] and Sójka et al. [32]. A similar profile of anthocyanins in raspberry (var. Polana) pomace was obtained in the study by Szymanowska et al. [33]. However, in the present study, five anthocyanins were identified for the raspberry pomace extract. In the cited work, only three anthocyanins were identified—cyanidin-3-sophoroside, which represented over 70% of the anthocyanin content, cyanidin-3-glucoside and cyanidin-3-rutinoside. The results are also consistent with an HPLC study of anthocyanins in freeze-dried Polana raspberries, where cyanidin-3-sophoroside has been described as a predominant compound, followed by cyanidin-3-glucoside [34]. HPLC analysis showed that substantial amounts of anthocyanins which have numerous health benefits [35] may be obtained through a green extraction procedure from berry fruit wastes.

### 2.5. TPC and Antioxidant Capacity Correlation

**Figure 3:**
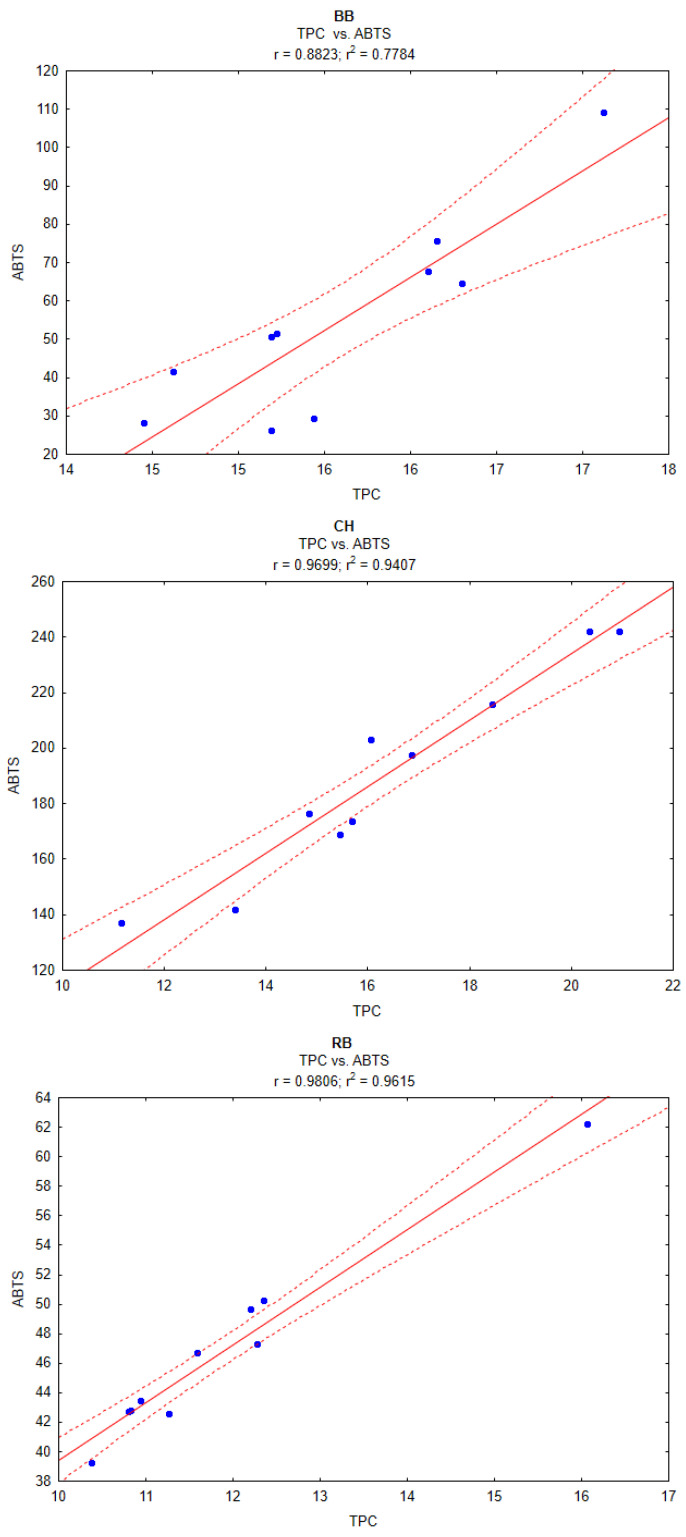
Graphs illustrating correlation between TPC and antioxidant capacity (ABTS) of pomace extracts. Dashed line marks 95% confidence interval.

To investigate the dependencies of pomace extracts’ antioxidant activity, the correlation between the TPC and antioxidant capacity, measured using an ABTS assay, was determined. A graphical illustration of the correlations is shown in Figure 3. The results obtained for different pomace extracts varied. In the cases of the CH and RB pomace extracts, the correlation between the TPC and antioxidant capacity was stronger—r = 0.9699 and r = 0.9806, respectively. BB pomace extract was characterized by a lower correlation, r = 0.8823. However, the obtained results are still quite high compared to, e.g., Babbar et al. [36], who studied the correlation between TPC and ABTS in extracts from different fruit residues. The overall correlation coefficient was equal to 0.70, but the researchers in this study used the seeds, pericarp or peels of litchi, kinnow and banana, not berry fruit pomaces. In the case of berry fruits, Dragović-Uzelac et al. [37] obtained a correlation coefficient value of r = 0.78–0.84 for the polyphenol-rich extracts of blueberries. Also, Rojas-Ocampo et al. [38] reported strong correlation between the TPC and antioxidant capacity, measured using DPPH and ABTS assays, for extracts from pulp and bagasse from blueberry, elderberry, blackberry and goldenberry. Similar results were presented by de Souza et al. [39] for blackberry, red raspberry, strawberry, blueberry and sweet cherry fruits. The high r correlation coefficients between the TPC and ABTS indicate that most antioxidants in the studied berry fruit pomaces were derived from polyphenolic compounds, with non-polyphenolic constituents, like ascorbic acid or carotenoids, having an only marginal influence.

## 3. Materials and Methods

### 3.1. Materials

Local farmers in Pulawy, Poland, supplied fresh raspberries (*Rubus idaeus* var. Polana) and chokeberries (*Aronia melanocarpa* var. Nero). The blackberries (*Rubus fruticosus* var. Brzezina) were obtained from the Horticulture-National Research Institute in Skierniewice, Poland, and the fruit came from a Rubus collection carried out as a part of the Polish Ministry of Agriculture and Rural Development’s targeted task of ex situ conservation of horticultural plant genetic resources. Juices were extracted from around 10 kg of fruits using a hydraulic press (HPL 14, Bucher Unipektin, Niederweningen, Switzerland) at a maximum pressure of 3 bar. Pomaces obtained from juice pressing were dried in a laboratory convection drier at 45 °C and 1.5 m/s airflow. Using a Rotronic Hygrolab C1 (Rotronic AG, Bassersdorf, Switzerland) hygrometer, the water content of dried pomaces was measured at 25 ± 0.3 °C. The water content values for all samples were lower than 0.4. The pomaces were then sieved to remove the seeds, and seedless pomaces were used as the study material.

### 3.2. Methods

#### 3.2.1. Ultrasound-Assisted Extraction

UAE was performed using the previously reported method [24] in a UP400S ultrasound processor (Hielscher Ultrasonics, Teltow, Germany) with an output power of 400 W. For every sample, two grams of pomace were placed in a falcon tube and filled with distilled water at a solid/liquid ratio of 1:15 shortly before extraction. To maintain a temperature below 45 °C, the falcon tube was immersed in an ice bath. An immersion, a thermometer was utilized to monitor the solid/solvent mixture. Following that, the extracts were filtered through a paper filter and analyzed.

#### 3.2.2. Total Polyphenol Content

TPC was determined using Folin–Ciocalteu reagent reaction, as described by Gao et al. [40]. In brief, 0.2 mL of diluted extracts, 0.4 mL Folin–Ciocalteu reagent, 4 mL distilled water and 2 mL of 15% sodium carbonate solution were combined in a test tube, stirred with a Vortex mixer and left in the dark for 60 min. After that, spectrophotometric analysis was carried out. The absorbance was measured at 765 nm with a Shimadzu UV-1650PC spectrophotometer (Shimadzu Corp., Kyoto, Japan). The calibration curve was prepared using working standard solutions of gallic acid at concentrations ranging from 50 mg to 250 mg/L. TPC values were given in milligrams of gallic acid equivalent (GAE) per gram of dried material.

#### 3.2.3. Antioxidant Capacity

Antioxidant capacity was determined using ABTS cation radical solution according to the method described by Re et al. [41]. Diluted extract (40 μL) was mixed with 4 mL ABTS working solution, stirred in a Vortex mixer and left in the dark for 8 min. After incubation, the absorbance of the samples was measured at 734 nm using a Shimadzu UV-1650PC spectrophotometer (Shimadzu Corp., Kyoto, Japan). Trolox standard curves were created using working standard solutions at concentrations ranging from 0 to 1125 μmol/L. Antioxidant capacity was given as μmol Trolox equivalent (TE) per gram of dried sample.

#### 3.2.4. High-Performance Liquid Chromatography

The anthocyanin content was examined using a HPLC device (Shimadzu Corp., Kyoto, Japan) equipped with a DAD detector and a Luna 5 μm C18 (2), 250 mm × 4.6 mm column with precolumn (Phenomenex, Torrance, CA, USA). Using the HPLC method in compliance with an earlier approach [42], the anthocyanin content in the extracts obtained under optimal and control conditions was ascertained. One milliliter per minute of isocratic flow was used for the analysis. The mobile phase consisted of a combination of water, acetonitrile and formic acid at a volumetric ratio of 830:70:100. At a wavelength of 520 nm, the findings were recorded. The compounds’ concentrations were measured in reference to a standard consisting of cyanidin-3-glucoside. For every sample, three separate analyses were conducted. With the help of the LabSolutions software (v. 5.106, Shimadzu Corp., Kyoto, Japan), the total anthocyanin content (TAC) was computed by adding the individual anthocyanins concentrations.

#### 3.2.5. Experimental Design

Experimental runs were carried out in triplicate in the conditions summarized in Table 5. The results were analyzed in order to fit appropriate mathematical models and equations explaining the influence of variables on the experimental responses according to the following formulas (Equation (7) for the two-factor interaction model and Equation (8) for the quadratic model):

(7) $$Y=\beta_{0}+\beta_{1}X_{1}+\beta_{2}X_{2}+\beta_{12}X_{1}X_{2}$$

(8) $$Y=\beta_{0}+\beta_{1}X_{1}+\beta_{2}X_{2}+\beta_{12}X_{1}X_{2}+\beta_{11}{X_{1}}^{2}+\beta_{22}{X_{2}}^{2}$$

where $\beta_{0}$—the constant coefficient; $\beta_{1}$, $\beta_{2}$—regression coefficients for the linear terms; $\beta_{11}$, ${\;\beta }_{22}$—regression coefficients for the quadratic terms; $\beta_{12}$—regression coefficient for the interaction terms; and $X_{1}$, $X_{2}$—coded values of independent variables.

#### 3.2.6. Statistical Analysis

The experiment was designed and analyzed in terms of model fitting (determination coefficients, lack-of-fit tests, ANOVA tests for fitted models and equation determination) and optimization using Design-Expert software (v. 22.0.2, Stat-Ease Inc., Minneapolis, MN, USA). The correlation between TPC and ABTS of extracts and statistical analysis (ANOVA, followed by post hoc Tukey’s test) of HPLC results were carried out in Statistica software (v. 13.3, Statsoft, Kraków, Poland). A *p* value of 0.05 was applied to determine the significant differences.

## 4. Conclusions

Blackberry, chokeberry and raspberry pomaces can be used as materials to extract polyphenols. By-products from the fruit-processing industry are still abundant in valuable bioactive compounds, and thus their extraction and further processing appear to be reasonable waste-management solutions. In order to achieve a fully green process, ultrasound-assisted extraction using water as an extractant can be considered. In the present study, it was shown that the quality of an extract depends on the amplitude of the ultrasound applied and the extraction time. Based on the conducted experiments, the extraction method was optimized in order to achieve the highest possible responses in terms of total polyphenol content and antioxidant capacity of water extracts. By applying response surface methodology, specific extraction conditions were calculated which should yield the predicted maximized response values. The mathematical models predicted values close to the actual obtained results with high accuracy, which is a proof RSM’s usability in optimization procedures. The extracts obtained in the most favorable conditions were also subjected to HPLC analysis of their anthocyanin content. The collected results demonstrate that pomaces can be a source of bioactive compounds; however, the extraction method has to be modified in order to provide efficient but also cost-effective and environmentally friendly process. The ultrasound-assisted extraction method can be considered as such a process, and its optimization confers maximized utilization of by-products.

## Figures and Tables

**Table 1:** ANOVA results for model fitting of TPC in pomace extracts.

Pomace	Model	R^2^	CV [%]	Model *p*-Value	Model F-Value	Lack of Fit *p*-Value
BB	Quadratic	0.8914	2.62	0.0461	6.57	0.1650
CH	Quadratic	0.9815	3.76	0.0015	42.35	0.5659
RB	2FI	0.7061	9.10	0.0490	4.80	0.0680

**Table 2:** ANOVA results for model fitting of antioxidant capacity in pomace extracts.

Pomace	Model	R^2^	CV [%]	Model *p*-Value	Model F-Value	Lack of Fit *p*-Value
BB	Quadratic	0.8991	22.68	0.0402	7.12	0.3987
CH	Quadratic	0.9747	4.65	0.0027	30.8	0.2730
RB	2FI	0.7354	8.73	0.0362	5.56	0.0684

**Table 3:** Optimal ultrasound-assisted extraction parameters with predicted vs. actual responses obtained.

Pomace	Optimum Ultrasound Amplitude [%]	Actual Ultrasound Amplitude Applied [%]	Optimum Extraction Time [min]	Predicted TPC [mg GAE/g]	Predicted ABTS [μmol TE/g]	Actual TPC [mg GAE/g]	Actual ABTS [μmol TE/g]
BB	98	95	5.00	17.04	106.14	17.60 ± 0.03	101.11 ± 1.01
CH	78	80	10.32	21.06	246.00	20.22 ± 0.14	252.26 ± 2.11
RB	90	90	11.75	16.83	66.62	16.21 ± 0.23	64.00 ± 1.55

**Table 4:** Anthocyanin content (mg/100 g fresh weight) of blackberry, chokeberry and raspberry pomace extracts, obtained under optimal UAE conditions.

Anthocyanin Name	Blackberry	Chokeberry	Raspberry
Cyanidin-3-galactoside	-	305.4 ± 6.5	-
Cyanidin-3-glucoside	107.9 ± 1.7	18.3 ± 0.1	31.6 ± 0.7
Cyanidin-3-arabinoside	1.4 ± 0.1	115.3 ± 1.9	-
Cyanidin-3-xyloside	8.1 ± 0.2	15.9 ± 0.4	-
Cyanidin-3-sophoroside	-	-	41.8 ± 0.5
Cyanidin-3-glucosylrutoside	-	-	20.2 ± 0.4
Cyanidin-3-rutinoside	-	-	19.0 ± 0.8
Pelargonidin-3-sophoroside	-	-	2.8 ± 0.1
TAC	117.4 ± 1.9 ^a^	454.8 ± 8.4 ^b^	115.3 ± 2.5 ^a^

TAC—total anthocyanin content; ‘-’ stands for not detected; ^a,b^—different letters in superscript indicate significantly different groups of results at *p* < 0.05.

**Table 5:** Experimental design—coded and actual values of ultrasound amplitude and extraction time.

Run	Ultrasound Amplitude X_1_		Extraction Time X_2_	
	Coded	Actual [%]	Coded	Actual
1	−1	30	−1	2
2	+1	80	−1	2
3	−1	30	+1	10
4	−1.414	80	+1	10
5	+1.414	19.75	0	6
6	0	90.25	0	6
7	0	55	−1.414	0.34
8	0	55	+1.414	11.66
9	0	55	0	6
10	0	55	0	6

## Data Availability

Data presented in the following study are available upon reasonable request from the corresponding author.
